# Regulation of Gene Expression with Thyroid Hormone in Rats with Myocardial Infarction

**DOI:** 10.1371/journal.pone.0040161

**Published:** 2012-08-01

**Authors:** Yue-Feng Chen, James V. Pottala, Nathan Y. Weltman, Xijin Ge, Olga V. Savinova, A. Martin Gerdes

**Affiliations:** 1 Department of Biomedical Sciences, New York College of Osteopathic Medicine of New York Institute of Technology, Old Westbury, New York, United States of America; 2 Sanford Research/University of South Dakota, Sioux Falls, South Dakota, United States of America; 3 Department of Mathematics and Statistics, South Dakota State University, Brookings, South Dakota, United States of America; AC Camargo Cancer Hospital, Brazil

## Abstract

**Introduction:**

The expression of hundreds of genes is altered in response to left ventricular (LV) remodeling following large transmural myocardial infarction (MI). Thyroid hormone (TH) improves LV remodeling and cardiac performance after MI. However, the molecular basis is unknown.

**Methods:**

MI was produced by ligation of the left anterior descending coronary artery in female SD rats. Rats were divided into the following groups: (1) Sham MI, (2) MI, and (3) MI+T4 treatment (T4 pellet 3.3 mg, 60 days release, implanted subcutaneously immediately following MI). Four weeks after surgery, total RNA was isolated from LV non-infarcted areas for microarray analysis using the Illumina RatRef-12 Expression BeadChip Platform.

**Results:**

Signals were detected in 13,188 genes (out of 22,523), of which the expression of 154 genes were decreased and the expression of 200 genes were increased in MI rats compared with Sham MI rats (false discovery rate (FDR) <0.05). Compared to MI rats, T4 treatment decreased expression of 27 genes and increased expression of 28 genes. In particular, 6 genes down-regulated by MI and 12 genes up-regulated by MI were reversed by T4. Most of the 55 genes altered by T4 treatment are in the category of molecular function under binding (24) and biological processes which includes immune system process (9), multi-organism process (5) and biological regulation (19) nonexclusively.

**Conclusions:**

These results suggest that altered expression of genes for molecular function and biological process may be involved in the beneficial effects of thyroid hormone treatment following MI in rats.

## Introduction

Left ventricular (LV) remodeling is a complicated process following a large transmural myocardial infarction (MI) involving both infarct and non-infarcted regions with changes in myocytes, interstitial matrix, and vasculature [1]. More than four hundred genes have been found to be involved in this process [2]. Targeted treatments that improve this process, such as β-blockers and angiotensin converting enzyme inhibitors, have led to great progress in alleviation of heart failure in post-MI patients. However, challenges remain since slow progression to heart failure continues.

Thyroid hormone is important in cardiovascular development and homeostasis. A number of patients experience a short period of low thyroid status (euthyroid sick syndrome) following MI and other serious medical conditions. Thus, thyroid hormone has been studied for the treatment of MI in different animal models. Results showed that thyroid hormone can reduce myocyte apoptosis and improve left ventricular remodeling and LV function after MI [3]–[8]. Although thyroid hormone receptors and some signal pathways have been proposed to mediate these effects, the underlying gene changes are still not clear.

With the development of microarray technology, the expression profiles of tens of thousands of genes can be studied simultaneously. We conducted this study using a female rat model treated with thyroxine (T4) for 4 weeks. The T4 dose was chosen based on our preliminary study showing a cardiac hypertrophic effect with a minor increase in the heart rate on the MI rats. Using Illumina's BeadChip microarray, the expression of 55 genes in the LV non-infarcted area have been found to be altered with thyroid hormone treatment and are reported here for the first time.

## Results

### General data

No animals died during the observation period. No difference was found in infarct area between MI and MI+T4 groups (Table 1), although three animals in each of MI and MI+T4 groups with infarct area <30% were excluded from this study. No difference was found in body weight before and 4 weeks after surgery among sham, MI and MI+T4 groups. MI caused a significant increase in total heart weight, heart weight/body weight ratio. T4 treatment after MI further increased heart weight, heart weight/body weight ratio and LV weight. No difference was detected in serum thyroid hormone levels at 4 weeks after MI. T4 treatment significantly increased serum T4 levels, but only slightly increased serum T3 levels which did not reach statistical significance at 4 weeks post-MI. (Table 1).

**Table 1:** General data.

Groups	N	Body Wt1 (g)	Body Wt2 (g)	Heart Wt (mg)	HW/BWt2 Ratio	LV Wt (mg)	Infarct Area (%)	Total T4 (ng/ml)	Total T3 (ng/ml)
Sham	9	232 (7)	261 (6)	793 (50)	3.0 (0.2)	577 (46)		21.1 (7.6)	1.5 (0.3)
MI	9	235 (7)	259 (10)	875 (67)*	3.4 (0.3)*	625 (39)	47.7 (6.1)	23.4 (6.3)	1.5 (0.6)
MI+T4	7	236 (6)	264 (9)	955 (70)**	3.6 (0.2)**	692 (61)**	46.3 (9.5)	43.2 (15.0)**^†^	1.9 (0.4)

Data presented as means (SD).

N = number of animals; Body Wt1 =  body weight before surgery; Body wt2  =  body weight at terminal study; Heart Wt  =  heart weight; HW/BWt2 ratio  =  Heart Wt/Body wt2 ratio; LV Wt  =  left ventricular weight; *, *p*<0.05, **, *p*<0.01 vs. Sham operated rats; †, *p*<0.05 vs. MI; ANOVA with Bonferroni's Multiple Comparison Test.

### LV morphological and functional changes

There was a significant increase in LV dimension and decrease in interventricular septal thickness as well as FS after MI. T4 treatment after MI improved FS but did not change the LV dimension and wall thickness significantly (Table 2). MI resulted in LVEDP elevation and –dp/dt reduction, as well as a tendency of +dp/dt reduction. T4 treatment tended to increase +dp/dt and heart rate, as well as decrease LVEDP and –dp/dt following MI, but did not reach statistical significance (Table 3).

**Table 2:** Echocardiographic data.

Groups	IVSd (mm)	IVSs (mm)	LVIDd (mm)	LVIDs (mm)	LVPWd (mm)	LVPWs (mm)	FS (%)
Sham	1.5 (0.4)	2.5 (0.5)	7.2 (0.7)	4.3 (0.6)	1.6 (0.3)	2.2 (0.4)	40.1 (4.4)
MI	1.3 (0.3)	1.8 (0.3)**	8.5 (0.8)**	6.7 (1.2)**	1.4 (0.4)	1.9 (0.5)	21.9 (8.1)**
MI+T4	1.4 (0.4)	2.1 (0.5)	8.5 (0.8)**	6.1 (1.0)**	1.5 (0.3)	2.3 (0.4)	27.4 (6.8)**

Data presented as means (SD). N, same as in Table 1.

IVSd and IVSs  =  interventricular septal thickness in end diastole and systole, respectively; LVIDd and LVIDs  =  left ventricular diastolic and systolic internal diameter, respectively; PWTd and PWTs  =  diastolic and systolic posterior wall thickness, respectively; **, *p*<0.01 vs. Sham operated rats; ANOVA with Bonferroni's Multiple Comparison Test.

**Table 3:** Hemodynamic data.

Groups	HR (beat/min)	LVSP (mmHg)	LVEDP (mmHg)	+dp/dt (mmHg/s)	−dp/dt (mmHg/s)
Sham	334 (36)	126 (10)	6.2 (1.7)	8783 (1648)	10035 (1936)
MI	343 (28)	117 (8)	9.3 (1.9)**	7648 (1437)	6779 (1480)**
MI+T4	371 (24)*	124 (12)	8.1 (2.1)	8603 (1649)	7851 (1811)*

Data presented as means (SD). N, same as in Table 1.

LVSP and LVEDP  =  left ventricular end-systolic and end-diastolic pressure, respectively; +dP/dt and −dP/dt  =  maximal rate of pressure development and decline, respectively; *, *p*<0.05, **, *p*<0.01 vs. Sham operated rats; ANOVA with Bonferroni's Multiple Comparison Test.

### Gene expression revealed by microarray

**Figure 1:**
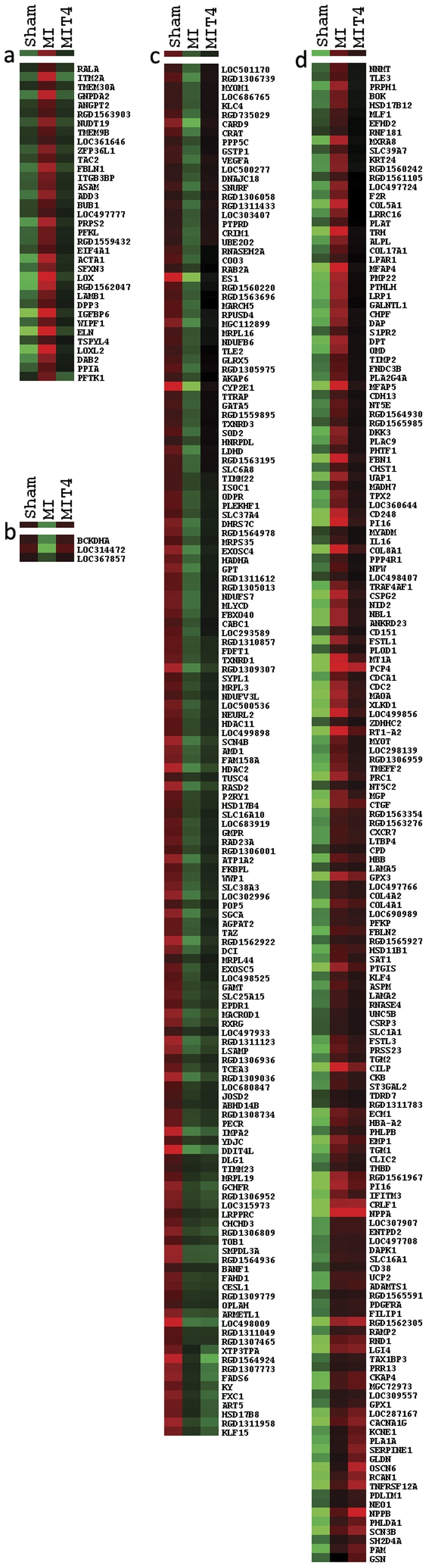
Gene clustering for 354 genes altered by MI. a. Genes whose expression was increased by MI but reduced or normalized by T4 treatment; b. Genes whose expression was decreased by MI but normalized by T4 treatment; c. Genes whose expression was reduced by MI but not reversed by T4 treatment; d. Genes whose expression was increased by MI but not reversed by T4 treatment.

Signals were detected in 13,188 genes (out of 22,523), of which 154 gene expressions were decreased and 200 gene expressions were increased in MI rats compared with Sham MI rats (false discovery rate (FDR) <0.05). There were 103 genes of these 354 genes with expressions increased/decreased ≥1.5 times over Shams after MI. Compared to MI rats, T4 treatment decreased the expression of 27 genes and increased the expression of 28 genes after MI (Figure 1a–d).

Most of these 354 genes altered by MI have multiple Gene Ontology (GO) annotation terms and functions (Table 4). Genes in the category of Cellular Component (CC) include Extracellular Region (56), Extracellular Region Part (36), Envelope (22) and Organelle (143). Genes in the category of Molecular Function (MF) include Binding (214), Catalytic Activity (103) and Antioxidant Activity (5). Genes in the category of Biological Process (BP) include Developmental Process (72), Cellular Process (157), Biological Regulation (119), Cellular Component Biogenesis (24), Reproductive Process (21), Reproduction (21), Cellular Component Organization (45), Metabolic Process (114) and Locomotion (12). There are still 87 genes with unknown functions.

**Table 4:** Functional annotation of 354 genes altered by MI (MI *vs*. Sham).

Functional category		Gene symbol
**Biological Process**		
	Developmental process	AGPAT2, ADAMTS1, KLF4, RND1, TIMP2, ACTA1, AMD1, ANGPT2, ASPM, CDH13, CDC2, F2R, COL4A1, COL5A1, COL8A1, CTGF, CKB, CSRP3, DKK3, DAB2, DLG1, ELN, EMP1, FBN1, FBLN1, GSN, GLDN, GPX1, GAMT, HBA-A2, HDAC11, HSD17B4, HSD11B1, KY, LAMA2, LAMA5, LAMB1, LGI4, LRP1, LOX, MGP, MLF1, NPPB, NEO1, NEURL2, NNMT, PTHLH, PAM, PPIA, PMP22, PLA2G4A, PLAT, PDGFRA, KCNE1, CABC1, PLOD1, RCAN1, RXRG, SERPINE1, HDAC2, SLC37A4, SOD2, TAZ, TXNRD1, THBD, TGM1, TGM2, TDRD7, TNFRSF12A, UNC5B, VEGFA, ZFP36L1
	Cellular process	NT5E, NT5C2, ART5, ATP1A2, BOK, CD38, DNAJC18, FKBPL, KLF4, NDUFS7, RAB2A, RAD23A, RPUSD4, RND1, ST3GAL2, TIMP2, TSPYL4, UAP1, WIPF1, WWP1, ACTA1, AMD1, ANGPT2, ANKRD23, ASPM, BCKDHA, BUB1, CDH13, CACNA1G, CHST1, CRAT, CDC2, F2R, COQ3, COL4A1, COL5A1, COL8A1, CTGF, CKB, CSRP3, CYP2E1, DAPK1, DAP, DPT, DAB2, DLG1, DCI, ENTPD2, ELN, EPDR1, EMP1, EXOSC5, FDFT1, FBLN1, FXC1, GSN, GLDN, GNPDA2, GPX1, GPX3, GSTP1, GAMT, GMPR, HBA-A2, HNRPDL, HDAC11, HADHA, HSD17B12, HSD17B4, HSD17B8, HSD11B1, ITGB3BP, IL16, KRT24, KY, LAMA2, LAMA5, LAMB1, LTBP4, LRPPRC, LGI4, LSAMP, LRP1, LPAR1, LOX, MLYCD, MGP, MT1A, MRPL16, MRPL3, MRPL44, MAOA, MLF1, NPPA, NPPB, NEO1, NEURL2, NID2, OMD, PTHLH, PAM, PPIA, PMP22, PECR, PFKL, PFKP, PLA2G4A, PRPS2, PLAT, PDGFRA, PLEKHF1, PHLDA1, KCNE1, CABC1, POP5, PLOD1, PTGIS, PPP5C, PTPRD, QDPR, RAMP2, RCAN1, RXRG, RNASEH2A, RNF181, SERPINE1, RGD1563903, HDAC2, LOC298139, BANF1, RGD1311612, SLC16A1, SLC37A4, SAT1, SMPDL3A, SOD2, TAZ, TXNRD1, TXNRD3, TRH, TCEA3, TOB1, TLE2, TLE3, TGM1, TGM2, TIMM22, TIMM23, TDRD7, TNFRSF12A, UBE2Q2, UNC5B, UCP2, RALA, VEGFA, ZFP36L1, ZDHHC2
	Biological regulation	AGPAT2, NT5E, AKAP6, ATP1A2, BOK, CD38, DDIT4L, GATA5, GCHFR, KLF15, KLF4, PDLIM1, RAB2A, RASD2, RT1-A2, RND1, TIMP2, TMEM9B, TAX1BP3, WWP1, ACTA1, ANGPT2, ASPM, BCKDHA, CDH13, CACNA1G, CILP, CARD9, CDC2, CXCR7, F2R, COL4A2, COL5A1, COL8A1, CTGF, CKB, CSRP3, CRIM1, DAPK1, DPT, DKK3, DAB2, DLG1, ENTPD2, ELN, EMP1, FNDC3B, FBLN2, FSTL3, GSN, GPX1, GAMT, HBA-A2, HNRPDL, HSD17B12, HSD17B8, HSD11B1, ECM1, IGFBP6, ITGB3BP, IFITM3, IL16, LAMA2, LAMA5, LAMB1, LTBP4, LRPPRC, LGI4, LRP1, LPAR1, MLYCD, MGP, MT1A, MFAP4, MAOA, NPPA, NPPB, NEO1, NEURL2, OMD, PTHLH, PPIA, PMP22, PFKL, PLA2G4A, PLAT, PDGFRA, PLEKHF1, PHLDA1, KCNE1, CABC1, PPP5C, P2RY1, RAMP2, RCAN1, RXRG, SERPINE1, HDAC2, BANF1, SLC37A4, SAT1, S1PR2, SOD2, TAC2, TXNRD1, THBD, TRH, TCEA3, TOB1, TLE2, TLE3, TGM2, TNFRSF12A, TUSC4, UBE2Q2, UNC5B, RALA, VEGFA, ZFP36L1
	Cellular component biogenesis	GCHFR, NDUFS7, TSPYL4, ACTA1, CDH13, ELN, GSN, GPX3, HBA-A2, HBB, LAMA5, LPAR1, LOX, MGP, NEURL2, PMP22, PFKL, PFKP, PPP5C, XTP3TPA, SLC1A1, SOD2, TAZ, TGM2
	Reproductive process	ADAMTS1, CD38, TIMP2, DLG1, FBLN1, FSTL3, GPX3, GAMT, HSD17B4, LAMB1, PTHLH, PAM, PPIA, PLA2G4A, PDGFRA, PHTF1, RAMP2, BANF1, THBD, TDRD7, VEGFA
	Reproduction	ADAMTS1, CD38, TIMP2, DLG1, FBLN1, FSTL3, GPX3, GAMT, HSD17B4, LAMB1, PTHLH, PAM, PPIA, PLA2G4A, PDGFRA, PHTF1, RAMP2, BANF1, THBD, TDRD7, VEGFA
	Cellular component organization	GCHFR, NDUFS7, RND1, TSPYL4, WIPF1, ACTA1, CDH13, CDC2, F2R, COL5A1, DPT, DAB2, DLG1, ELN, FBLN1, FXC1, GSN, GPX1, GPX3, HBA-A2, HBB, HDAC11, HSD17B12, KY, LAMA2, LAMA5, LAMB1, LRP1, LPAR1, LOX, MGP, NEURL2, PMP22, PFKL, PFKP, PDGFRA, PPP5C, HDAC2, XTP3TPA, SLC1A1, SOD2, TAZ, TGM2, UNC5B, RALA
	Metabolic process	NT5E, NT5C2, ADAMTS1, ART5, ATP1A2, CD38, DNAJC18, FKBPL, NDUFS7, RAD23A, RPUSD4, ST3GAL2, UAP1, WWP1, AMD1, ALPL, ANKRD23, BCKDHA, BUB1, CHST1, CPD, CRAT, CDC2, F2R, COQ3, COL5A1, CTGF, CKB, CYP2E1, DAPK1, DLG1, DCI, ENTPD2, EXOSC5, FDFT1, FAHD1, GNPDA2, GPT, GPX1, GPX3, GSTP1, GAMT, GMPR, HNRPDL, HDAC11, HADHA, HSD17B12, HSD17B4, HSD17B8, HSD11B1, ITGB3BP, ISOC1, LRPPRC, LRP1, LPAR1, LOX, LOXL2, MLYCD, MRPL16, MRPL3, MRPL44, MAOA, MLF1, NPPA, NPPB, NID2, PTHLH, PAM, PPIA, PECR, PFKL, PFKP, PLA1A, PLA2G4A, PHLPB, PRPS2, PLAT, PDGFRA, PHLDA1, CABC1, POP5, PLOD1, PTGIS, PRSS23, PPP5C, PTPRD, QDPR, RXRG, RNASEH2A, RNF181, RGD1563903, DPP3, HDAC2, LOC298139, BANF1, RGD1311612, RGD1306809, SLC16A1, SLC37A4, SAT1, SMPDL3A, SOD2, TAZ, TXNRD1, TXNRD3, TCEA3, TLE2, TLE3, TGM1, TGM2, UBE2Q2, VEGFA, ZFP36L1, ZDHHC2
	Locomotion	ATP1A2, CDH13, COL5A1, CTGF, GPX1, IL16, LAMA5, PLAT, CABC1, SLC37A4, TNFRSF12A, VEGFA
**Cellular Component**		
	Extracellular region	ADAMTS1, ART5, ST3GAL2, TIMP2, ANGPT2, CDH13, CILP, F2R, COL4A1, COL4A2, COL5A1, CTGF, CRIM1, DKK3, ENTPD2, ELN, EPDR1, ES1, FBN1, FBLN1, FSTL1, FSTL3, GSN, GPX3, HSD17B12, ECM1, IGFBP6, LAMA2, LAMA5, LAMB1, LTBP4, LOX, MGP, MFAP5, NPPA, NPPB, NBL1, NPW, NID2, OMD, PTHLH, PI16, PAM, PPIA, PLA1A, PLAT, KCNE1, PTGIS, PRSS23, RNASE4, SERPINE1, SMPDL3A, TAC2, THBD, TRH, VEGFA
	Extracellular region part	ADAMTS1, TIMP2, CDH13, CILP, COL4A1, COL4A2, COL5A1, TGF, ENTPD2, ELN, ES1, FBN1, FBLN1, FSTL3, GSN, GPX3, SD17B12, ECM1, IGFBP6, LAMA2, LAMA5, LAMB1, LTBP4, LOX, MGP, MFAP5, NPPB, NID2, OMD, PAM, PLAT, KCNE1, PTGIS, SMPDL3A, THBD, VEGFA
	Envelope	AKAP6, GCHFR, NDUFB6, NDUFS7, CRAT, CHCHD3, DCI, FXC1, FAHD1, HADHA, HSD17B8, HSD11B1, LDHD, LRPPRC, MRPL19, MAOA, SFXN3, SLC25A15, SOD2, TIMM22, TIMM23, UCP2
	Organelle	AGPAT2, AKAP6, ADAMTS1, ATP1A2, CD38, GATA5, GCHFR, KLF15, KLF4, MACROD1, NDUFB6, NDUFS7, PDLIM1, PFTK1, PCP4, RAB2A, RAD23A, RPUSD4, RGD735029, ST3GAL2, TIMP2, TPX2, TRAF4AF1, TSPYL4, TAX1BP3, UAP1, WIPF1, ABHD14B, ACTA1, ADD3, ASAM, ANKRD23, ASPM, BCKDHA, BUB1, CHST1, CESL1, CPD, CRAT, CDC2, CHPF, F2R, COQ3, CHCHD3, CKB, CSRP3, CYP2E1, CKAP4, DAPK1, DAB2, DLG1, DCI, ELN, EPDR1, ES1, FDFT1, FNDC3B, FILIP1, FSTL3, FXC1, FAHD1, GSN, GLRX5, GPX1, GSTP1, HBA-A2, HNRPDL, HDAC11, HADHA, HSD17B12, HSD17B4, HSD17B8, HSD11B1, IGFBP6, ITGB3BP, IFITM3, ISOC1, KRT24, KLC4, LDHD, LRPPRC, LRP1, LOX, MLYCD, MGP, MT1A, MRPL16, MRPL19, MRPL3, MRPL44, MRPS35, MAOA, MLF1, NPPA, NPPB, NUDT19, LOC683919, PTHLH, PAM, PPIA, PECR, PLA2G4A, PLAT, PLEKHF1, PHLDA1, KCNE1, CABC1, POP5, PLOD1, PTGIS, PRSS23, PPP5C, PHTF1, QDPR, RCAN1, RXRG, RNASEH2A, SFXN3, RGD1563903, HDAC2, LOC298139, RGD1307465, RGD1565591, BANF1, SNURF, SLC16A1, SLC25A15, SLC37A4, SOD2, SYPL1, TAZ, TXNRD1, TRH, TCEA3, TLE2, TLE3, TGM2, TIMM22, TIMM23, TDRD7, UCP2, VEGFA, ZFP36L1
**Molecular Function**		
	Binding	NT5E, OPLAH, AKAP6, ADAMTS1, ATP1A2, BOK, CD151, CD38, DNAJC18, EFHD2, FBXO40, FKBPL, GATA5, GCHFR, LOC500277, KLF15, KLF4, NDUFS7, PDLIM1, PFTK1, PCP4, RAB2A, RAD23A, RASD2, RGD1559432, RPUSD4, RT1-A2, RND1, RGD735029, SH2D4A, TIMP2, TPX2, TRAF4AF1, TAX1BP3, TTRAP, UAP1, WIPF1, WWP1, ACTA1, ADD3, AMD1, ALPL, ANGPT2, MGC72973, BCKDHA, BUB1, CDH13, CACNA1G, CPD, CARD9, CDC2, CXCR7, CLIC2, CHPF, F2R, CHCHD3, COL4A1, COL4A2, COL5A1, CTGF, CKB, CSRP3, CRIM1, CYP2E1, CRLF1, DAPK1, DAB2, DLG1, DCI, ENTPD2, ELN, EPDR1, EIF4A1, EXOSC4, EXOSC5, FDFT1, FBN1, FBLN1, FBLN2, FSTL1, FSTL3, FXC1, FAHD1, GSN, LOC287167, GPT, GPX1, GPX3, GSTP1, GAMT, GMPR, HBA-A2, HBB, HNRPDL, HDAC11, HADHA, HSD17B12, HSD17B4, HSD17B8, HSD11B1, IMPA2, IGFBP6, IL16, KLC4, LDHD, LAMA2, LAMA5, LAMB1, LTBP4, LRPPRC, LGI4, LSAMP, LRP1, LPAR1, LOX, LOXL2, MGP, MARCH5, MT1A, MFAP4, MRPL16, MRPL44, MAOA, MLF1, MYOT, NPPA, NPPB, NEO1, NEURL2, NBL1, NPW, NID2, NUDT19, OMD, PTHLH, PAM, PPIA, PECR, PFKL, PFKP, PLA2G4A, PRPS2, PDGFRA, PLEKHF1, PHLDA1, KCNE1, CABC1, POP5, PLOD1, PTGIS, PPP4R1, PPP5C, PRC1, P2RY1, QDPR, RAMP2, RCAN1, RXRG, RNASEH2A, RNASE4, RNF181, SERPINE1, SFXN3, RGD1306959, DPP3, LOC690989, HDAC2, LOC298139, XTP3TPA, RGD1565591, BANF1, RGD1560220, RGD1311612, SCN4B, SCN3B, SLC1A1, SLC16A1, SLC25A15, SLC38A3, SLC39A7, SLC6A8, SAT1, SMPDL3A, SOD2, TXNRD1, THBD, TRH, TCEA3, TOB1, TLE2, TLE3, TGM1, TGM2, TIMM22, TIMM23, TDRD7, TNFRSF12A, TUSC4, UNC5B, UCP2, RALA, VEGFA, ZFP36L1, ZDHHC2
	Catalytic activity	AGPAT2, NT5E, NT5C2, OPLAH, ADAMTS1, ART5, ATP1A2, CD38, DNAJC18, NDUFS7, PFTK1, RPUSD4, ST3GAL2, UAP1, WWP1, ABHD14B, AMD1, ALPL, BCKDHA, BUB1, CHST1, CESL1, CPD, CRAT, CDC2, CHPF, COQ3, CKB, CYP2E1, DAPK1, DCI, ENTPD2, ES1, EIF4A1, EXOSC5, FDFT1, FAHD1, GNPDA2, GPT, GPX1, GPX3, GSTP1, GAMT, GMPR, HDAC11, HADHA, HSD17B12, HSD17B4, HSD17B8, HSD11B1, IMPA2, ISOC1, KLC4, LDHD, LOX, LOXL2, MLYCD, MRPL44, MAOA, NUDT19, LOC683919, PAM, PPIA, PECR, PFKL, PFKP, PLA1A, PLA2G4A, PHLPB, PRPS2, PLAT, PDGFRA, CABC1, POP5, PLOD1, PTGIS, PRSS23, PPP5C, PTPRD, QDPR, RNASEH2A, RNASE4, RNF181, SERPINE1, DPP3, HDAC2, RGD1306739, LOC298139, XTP3TPA, RGD1311612, RGD1306809, SAT1, SMPDL3A, SOD2, TAZ, TXNRD1, TXNRD3, TLE2, TGM1, TGM2, TUSC4, UBE2Q2, ZDHHC2
	Antioxidant activity	GPX1, GPX3, SOD2, TXNRD1, TXNRD3
**Unknown**		LOC314472, LOC303407, RGD1311433, LOC367857, LOC501170, MYOM1, RGD1306058, LOC686765, RGD1305975, MGC112899, RGD1563195, RGD1559895, RGD1564924, RGD1563696, LOC293589, RGD1305013, FADS6, RGD1307773, RGD1562922, RGD1564978, DHRS7C, LOC500536, SGCA, RGD1309307, RGD1306001, LOC498525, NDUFV3L, LOC302996, LOC499898, FAM158A, RGD1310857, RGD1306936, RGD1311123, LOC497933, JOSD2, LOC680847, RGD1309036, RGD1311958, RGD1308734, LOC315973, RGD1306952, RGD1564936, LOC498009, ARMETL1, RGD1309779, RGD1311049, ITM2A, TMEM30A, LOC361646, RGD1562047, LOC497777, RGD1560242, LRRC16, GALNTL1, QSCN6, CD248, PRPH1, LOC497724, RGD1561105, MXRA8, COL17A1, RGD1565985, LOC499856, PLAC9, CSPG2, LOC498407, XLKD1, MADH7, LOC360644, RGD1564930, RGD1561967, MYADM, TMEFF2, CDCA1, LOC497766, RGD1563354, RGD1311783, RGD1563276, LOC309557, PRR13, RGD1562305, RGD1565927, LOC497708, LOC307907

Most of the 55 genes altered by T4 treatment are in the category of MF under Binding (24) and BP which includes Immune System Process (9), Multi-organism Process (5) and Biological Regulation (19). 5 of these 55 genes are in the category of CC under Extracellular Region Part. However, functions of 21 of these 55 genes remain unknown (Table 5).

**Table 5:** Functional annotation of 55 genes altered by T4 treatment (MI+T4 *vs*. MI).

Functional category	Gene symbol
**Biological Process**	
Immune system process	OAS1A, OASL2, ABCC9, RT1-M6-2, RT1-N2, CARD9, CXCL12, RT1-S3, IRF7
Multi-organism process	OAS1A, ABCC9, CARD9, IRF7, MX1
Biological regulation	OAS1A, RASA1, TMEM9B, ANGPT2, BCKDHA, CARD9, CXCL12, FGF1, GSN, ITGB3BP, IRF7, IRF9, NFIX, PLAG1, YWHAQ, SPOCK2, SPRED1, RALA, ZFP36L1
**Cellular Component**	
Extracellular region part	CXCL12, FGF1, GSN, MYOC, SPOCK2
**Molecular Function**	
Binding	OAS1A, OASL2, ABCC9, KCNIP2, PFTK1, RASA1, RGD735029, ADD3, ANGPT2, BCKDHA, BUB1, CARD9, CXCL12, FGF1, GSN, IRF7, IRF9, MYOC, MX1, NFIX, PLAG1, PAIP1, YWHAQ, RGD1559924, SPOCK2, SF3B3, SPRED1, RALA, ZFP36L1
**Unknown**	LOC688103, RGD1304646, LOC310926, TNFSF5IP1, ITM2A, HLA-DMB, MRPL52, TMEM30A, GNPDA2, LOC361646, ISG12(B), RGD1564163, IFI27L, RGD1562052, LOC314472, RGD1311874, RGD1310093, TYKI, LOC303407, RGD1311433, SLC43A3

There were 6 genes down-regulated by MI but up-regulated by T4 treatment and 12 genes up-regulated by MI but down-regulated by T4 treatment (Table 6).

**Table 6:** Fold changes in genes whose expression altered by MI but reversed by T4 treatment.

Gene Symbol	MI vs. Sham	MI+T4 vs. MI	MI+T4 vs. Sham
CARD9	0.63	1.35	0.85
RGD735029	0.76	1.19	0.91
LOC314472	0.67	1.52	1.01
BCKDHA	0.78	1.30	1.01
LOC303407	0.83	1.16	0.97
RGD1311433	0.78	1.22	0.96
ZFP36L1	1.36	0.77	1.05
PFTK1	1.22	0.76	0.92
ITGB3BP	1.30	0.82	1.06
BUB1	1.21	0.86	1.04
ADD3	1.41	0.78	1.10
ANGPT2	1.21	0.84	1.01
ITM2A	1.44	0.68	0.97
LOC361646	1.16	0.88	1.02
RALA	1.19	0.83	0.99
TMEM9B	1.21	0.83	1.01
GNPDA2	1.49	0.68	1.02
TMEM30A	1.19	0.84	0.99

### Validation of microarray results by RT-PCR

The expression of five selected genes (COL8A1, GPX3, NPPA, IFI27L and βMHC) was verified by quantitative RT-PCR. The fold changes in the expression levels of target genes revealed by RT-PCR were similar to or higher than those determined by microarray. In each gene, RT-PCR confirmed the relative differences between groups observed in microarray (Table 7).

**Table 7:** Fold changes in the expression of selected genes by RT-PCR.

Gene Symbol	Fold Changes (MI *vs*. Sham)		Fold Changes (MI+T4 *vs*. MI)	
	Microarray	RT PCR	Mcroarray	RT PCR
COL8A1	3.1	6.5	0.66	0.77
GPX3	2.0	2.3	0.93	1.2
NPPA	4.22	11.8	1.01	0.86
lFI27L	0.94	0.8	1.46	2.4
βMHC	0.99	1.80	1.00	0.83

### Angiopoietin-2 expression

**Figure 2:**
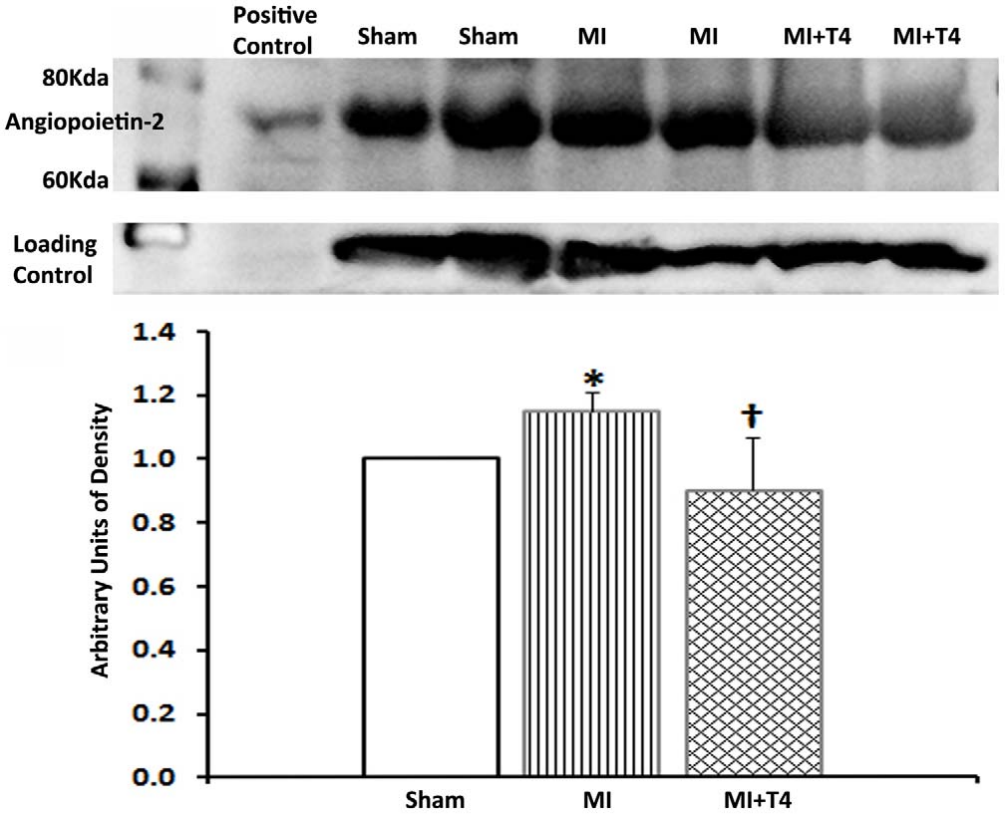
Representative Western blot and densitometry for Angiopoietin-2 expression in non-infarcted myocardium. Results are mean (SD) with n = 4 rats per group. *, *p*<0.05 vs. Sham operated group; †, *p*<0.05 vs. MI group; ANOVA with Bonferroni's Multiple Comparison Test.

Angiopoietin-2 expression was increased in non-infarcted myocardium after MI but decreased with T4 treatment as examined by Western blotting (Figure 2), which is consistent with microarray findings.

## Discussion

In this study, 354 genes were found to have altered expression in the non-infarcted myocardium 4 weeks after myocardial infarction. 103 of these 354 genes were found to have ≥1.5 time changes in the expression. 55 of these 354 genes were found to show altered expression with T4 treatment. Most of these 354 genes have multiple functions in the categories of Biological Process, Molecular Function and Cellular Component.

Left ventricular remodeling after MI is a very complicated process. Stanton et al [2] reported that more than 400 different gene expressions were altered in the remodeling myocardium at 2, 4, 8, 12 and 16 weeks post MI, with both temporal and spatial changes. The expression of 101 genes in the interventricular septum tissue was significantly changed at 4 weeks post MI. Most of these genes were involved in metabolism, cellular component synthesis, gene expression and intracellular communication. With the Illumina microarray, more genes in the non-infarcted myocardium were detected with altered expression at 4 weeks after MI in the present study.

Thyroid hormone has important effects on the cardiovascular system, including cardiovascular development and homeostasis under physiological and pathological status. Thyroid hormone has been shown to increase cardiac contractility, induce cardiac hypertrophy and angiogenesis, reduce apoptosis, improve LV remodeling and function in different animal models [4]–[6], [9]. Thyroid hormone induced cardiac hypertrophy is mediated by altered expression of a number of genes. Using cDNA microarray, Amamson et al [10] reported that T3 treatment for 3 days led to increased expression of 27 genes and decreased expression of 9 genes in cultured hypothyroid fetal rat myocytes. De et al [11] showed that T3 treatment of rats for 15 days resulted in upregulation of 11 genes and downregulation of 26 genes, which related to metabolism, cytoskeletal/matrix protein, hormone/growth factor, Ca^2+^-channel proteins and the proteins related to receptor transcription. Our microarray results show that T4 treatment decreased the expression of 27 genes and increased the expression of 28 genes after MI. Such gene profile is quite different from what Amamson et al and De et al have found. Since Amamson et al used cultured hypothyroid myocytes and De et al used hyperthyroid rats, we believe thyroid hormone may have different actions on gene expression during the late remodeling process after MI. Post-MI LV remodeling is characterized by an increased expression of fetal gene program, such as β myosin heavy chain (βMHC) gene. Ojamaa et al [3] reported that treatment with high dose T3 for 2 or 3 weeks post-MI resulted in a decrease in βMHC gene expression. Pantos et al [5] have shown that T3 and T4 combined treatment for 13 weeks after MI reversed βMHC expression. Our microarray did not show any difference in βMHC expression in non-infarcted myocardium at 4 weeks after MI with or without T4 treatment. RT-PCR showed a tendency of increased βMHC expression after MI and decreased βMHC expression with T4 treatment but did not reach statistical significance. Both Ojamaa and Pantos used T3 in their study, but in the present study serum T3 did not increase significantly. This might explain the difference in the findings as βMHC gene is T3 responsive. When compared, the selected genes examined with both microarray and RT-PCR in current study, RT-PCR has shown a higher fold change in expression when a difference was detected with microarray, indicating that RT-PCR is more sensitive than microarray and the Illumina Microarray Platform used in this study might not be sensitive enough in detecting some post-MI fetal gene program changes in rats.

There were 6 genes (CARD9, BCKDHA, etc.) down-regulated by MI but up-regulated by T4 treatment and 12 genes (Zfp36l1, ADD3, Angpt 2, etc.) up-regulated by MI but down-regulated by T4 treatment (Table 6). The CARD9 gene encodes a caspase recruitment domain-containing protein which interacts with BCL10 and activates the NF-*κ*B signaling pathway [12]. NF-*κ*B is a ubiquitously expressed redox-sensitive transcription factor that can respond to a large variety of stimuli including cytokines and stress, and regulate the expression of a great diversity of genes controlling cell survival and apoptosis in different cell types and at different times [13]. In the heart, NF-*κ*B has been shown to protect adult cardiac myocyte against ischemia-induced apoptosis in acute MI [14], as well as mediate hypoxia/reoxygenation-promoted angiogenesis in non-infarcted/border zone after MI [15]. The Zfp36l1 gene encodes a zinc finger-containing mRNA binding protein. It is important in both extraembryonic and intraembryonic angiogenesis. It is also a negative regulator of VEGF –A production via influencing the V*egf* mRNA translation [16]. Angiopoietin-1 (Ang-1) and angiopoietin-2 (Ang-2) are two isoforms of angiopoietins being identified as new angiogenesis regulators. They bind to and act as agonist (Ang-1) or antagonist (Ang-2) of the vascular endothelial receptor tyrosine kinase, Tie-2, and promote (Ang-1) or disrupt angiogenesis (Ang-2) [17], [18]. Sandhu et al [19] have reported that Ang-1 expression was decreased, whereas Ang-2 expression was increased in both the infarct and peri-infarct zone at 24 hours after MI in rats. While Ang-1 expression had returned to normal at 1 week post-MI, Ang-2 expression remained elevated in the infarct zone but returned to normal by 6 weeks after MI. In a diabetic MI mouse model, shifting the Ang-2-to-Ang-1 ratio to favor Ang-1 has been shown to reduce myocardial apoptosis and infarct size, as well as enhance angiogenesis. Shifting the Ang-2-to-Ang-1 ratio to favor Ang-2 resulted in a significant increase in infarct size [20]. The up-regulated CARD9 expression as well as down-regulated Zfp36l1 and Ang-2 expressions by T4 treatment in the present study might contribute to the pro-angiogenic effects of thyroid hormone.

The BCKDHA gene encodes the E1-alpha subunit of the branched-chain alpha-keto acid dehydrogenase complex which functions in the catabolism of branched-chain amino acids [21]. Add3 gene encodes a cytoskeletal actin filament (F-actin) capping protein which binds to the fast-growing ends of F-actin, such binding stabilizes/polymerizes the cytoskeleton, prevents the loss or addition of actin subunits and preserve the cell morphology [22]. High levels of Add3 could inhibit cellular growth, cellular shape changes and contractile force [23]. Thus, the up-regulated BCKDHA and down-regulated Add3 expression by T4 treatment in this study might explain the hypertrophic effects of thyroid hormone.

It should be pointed out that current study only focused on the cardiac gene expression changes at 4 weeks post MI, but the post-MI LV remodeling is continuous and temporal differences in cardiac gene expression might exist. The observed effects on cardiac gene expression following T4 treatment might be partially due to its effects on the heart rate and peripheral vascular resistance which cannot be excluded by the present study. Other molecular mechanisms involved in thyroid hormone effects cannot be underestimated, such as non-genomic actions and changes in microRNA expression [24]–[27].

## Materials and Methods

### Experimental design

Adult female Sprague-Dawley rats aged 12 weeks were used in this study. Myocardial infarction was induced by ligation of the left descending coronary artery. Immediately following the surgery, survivors were randomly assigned to the following groups: (1) MI group (n = 9); (2) MI + T4 group (n = 7); and (3) Sham MI group (n = 9). Shams were produced with an identical procedure except the suture was tied loosely around the coronary artery. T4 pellets (3.3 mg, 60 days sustained release. Innovative Research of America, Sarasota, FL) were implanted subcutaneously in the MI+T4 animals shortly after surgery. A placebo pellet was implanted in the MI and sham-operated animals at the same time. Animals were housed two per cage and kept on a 12 h light/dark cycle with food and water provided ad libitum. All experiments and protocols were performed in accordance with the Guide for the Care and Use of Laboratory Animals (US Department of Health, Education, and Welfare, Department of Health and Human Services, NIH Publication 85–23), and approved by the Animal Care and Use Committee of Sanford Research/University of South Dakota (Approval ID 18-08-09-12D).

### Echocardiographic measurements

Echocardiography was performed in each animal before it was euthanized using a Visualsonics Vevo 660 imaging system (Toronto, Canada) as described previously [4], [28]. Briefly, rats were anesthetized with isoflurane (1.5%) and two dimensional echocardiograms were obtained from short-axis views of the left ventricle at the level of the papillary muscle tips. Two-dimensionally targeted M-mode echocardiograms were used to measure the wall thickness and LV dimensions in systole and diastole.

### Cardiac hemodynamic measurements

Rats were anesthetized using isoflurane (1.5%), LV hemodynamics were obtained by catheterization of the right carotid artery using a Millar Micro-tip catheter (Millar Instruments; Houston, TX) as described before [29]. Data were recorded using a Millar Pressure-Volume System (model MPVS400; Millar Instruments; Houston, TX). The following data were collected: heart rate (HR), LV peak systolic pressure (LVSP), LV end-diastolic pressure (LVEDP), and positive/negative change in pressure over time (dp/dt).

### Tissue collection

Animals were sacrificed 4 weeks after surgery under anesthesia. The chest was opened and the heart was arrested by injection of potassium chloride via the inferior vena cava. The heart was quickly removed and immediately placed in ice-cold PBS. The heart was then cannulated with an 18 gauge gavage needle through the aorta, perfused with ice cold PBS, and subsequently trimmed, blotted, and weighed. The LV plus septum and the right ventricle (RV) were dissected and weighed. The LV was then cut into 3 pieces transversely, perpendicularly to the LV long axis. The basal slice was frozen in liquid nitrogen and stored in −80°C until used for RNA preparation. The middle slice was fixed in 4% paraformaldehyde overnight, embedded in paraffin, and sectioned (5 µm) for histological evaluation.

### Masson's trichrome staining

Paraformaldehyde-fixed transverse tissue slides were stained with Masson's trichrome. High resolution images were obtained through Aperio Scanscope software (Sanford-Burnham Institute, La Jolla, CA). Infarct size was estimated by measuring the percentage of endocardial and epicardial circumferences replaced by infarcted tissue using the following formula: Infarct size (%)  =  [(Infarcted tissue outer length + Infarcted tissue inner length)/(Left ventricular transversal epicardial circumference + Left ventricular transversal endocardial circumference)] ×100% [30]. MI animals with infarct size less than 30% or larger than 60% were excluded from the study. Four animals from each group were then randomly selected for genomic analysis.

### Microarray analysis

R/Bioconductor software was used for analysis of Illumina RatRef-12 Expression BeadChip microarrays [31], [32]. Four samples from each of the three groups were randomly assigned to one of the twelve channels on the BeadChip, and a second BeadChip was used as a technical replicate. The 24 arrays were then preprocessed using a variance stabilizing transformation similar to log2, but which specifically considered the bead replicates within an array [33]. Robust spline normalization was used to make the arrays comparable [34]. A critical level of 0.05 was used to make present calls, if a gene was not detected on any array it was dropped from further analysis. A common correlation between the technical replicates was used to increase the precision in the gene-wise variances [35]. An empirical Bayes one-way ANOVA method with a moderated F-statistic was used to determine differentially expressed genes [36]. The False Discovery Rate (FDR) was controlled at 5% [37]. Microarray data were publicly stored at Gene Expression Omnibus (GEO) per MIAME guidelines (Accession number: GSE35088) [38].

### Gene clustering

Gene cluster analysis was done using Cluster 3.0 software [39], [40]. After applying log transformation of the data, hierarchical clustering was applied using complete linkage clustering. The result was visualized using TreeView software [39].

### Functional annotation

Functional profiling of gene lists was performed using DAVID [41], [42], a web-based toolset. Genes were classified into three types of knowledge based on Gene Ontology (GO) annotation, that is: cellular component (CC), biological process (BP) and molecular function (MF).

### Real-time quantitative PCR for gene expression

Selected genes identified by microarray results were verified by real-time PCR performed by the Genomic-Microarray/qPCR Core at Sanford-Burnham Institute for Medical Research (La Jolla, CA). Briefly, RNA from the basal part of left ventricle was extracted using the PureLink™ RNA Mini Kit (Invitrogen, Carlsbad, CA). Oligo (dT) primed cDNA synthesis was performed using Superscript III (Invitrogen, Carlsbad, CA). Gene expression was measured using the TaqMan Gene Expression Assay kit using validated primers (Applied Biosystems, Foster City, CA).

### Western blotting

Protein was extracted from frozen basal region of LV tissue samples in RIPA buffer with protease cocktail inhibitor (EMD Biosciences Inc., San Diego, CA) and quantified with a BCA protein assay. Samples (150 µg) were loaded onto SDS-PAGE gels and transferred onto PVDF membranes and detected by angiopoietin-2 antibody (Santa Cruz Biotechnology, Inc., Santa Cruz, CA). Resultant bands were visualized by chemiluminescence and quantified using a KODAK 4000MM Digital Imaging System (Carestream Health, Inc., New Haven, CT).

### ELISA

Blood samples were collected from opened chests after hearts were removed and separated into serum aliquots by centrifugation and stored at −70°C until assayed. Serum total T3 and T4 levels were measured using ELISA kits according to the manufacturers' specification. The T3 and T4 kits were obtained from Monobind Inc. (human kit, Lake Forest, CA).

### Statistical analysis

All data except microarray data are expressed as means (SD). For multiple comparisons, one-way ANOVA followed by Bonferroni post-hoc test was applied to examine significant differences between groups. A value of P<0.05 was considered statistically significant.

## References

[1] Pfeffer MA, Braunwald E (1990) Ventricular remodeling after myocardial infarction. Experimental observations and clinical implications. Circulation 81: 1161–1172. 2138525 10.1161/01.cir.81.4.1161

[2] Stanton LW, Garrard LJ, Damm D, Garrick BL, Lam A, et al (2000) Altered patterns of gene expression in response to myocardial infarction. Circ Res 86: 939–945. 10807865 10.1161/01.res.86.9.939

[3] Ojamaa K, Kenessey A, Shenoy R, Klein I (2000) Thyroid hormone metabolism and cardiac gene expression after acute myocardial infarction in the rat. Am J Physiol Endocrinol Metab 279: E1319–1324. 11093920 10.1152/ajpendo.2000.279.6.E1319

[4] Chen YF, Kobayashi S, Chen J, Redetzke RA, Said S, et al (2008) Short term triiodo-L-thyronine treatment inhibits cardiac myocyte apoptosis in border area after myocardial infarction in rats. J Mol Cell Cardiol 44: 180–187. 17964598 10.1016/j.yjmcc.2007.09.009 PMC2235814

[5] Pantos C, Mourouzis I, Markakis K, Tsagoulis N, Panagiotou M, et al (2008) Long-term thyroid hormone administration reshapes left ventricular chamber and improves cardiac function after myocardial infarction in rats. Basic Res Cardiol 103: 308–318. 18274800 10.1007/s00395-008-0697-0

[6] Henderson KK, Danzi S, Paul JT, Leya G, Klein I, et al (2009) Physiological replacement of T3 improves left ventricular function in an animal model of myocardial infarction-induced congestive heart failure. Circ Heart Fail 2: 243–252. 19808346 10.1161/CIRCHEARTFAILURE.108.810747

[7] Kalofoutis C, Mourouzis I, Galanopoulos G, Dimopoulos A, Perimenis P, et al (2010) Thyroid hormone can favorably remodel the diabetic myocardium after acute myocardial infarction. Mol Cell Biochem 345: 161–169. 20730619 10.1007/s11010-010-0569-4

[8] Forini F, Lionetti V, Ardehali H, Pucci A, Cecchetti F, et al (2011) Early long-term L-T3 replacement rescues mitochondria and prevents ischemic cardiac remodelling in rats. J Cell Mol Med 15: 514–524. 20100314 10.1111/j.1582-4934.2010.01014.x PMC3922373

[9] Breisch EA, White FC, Hammond HK, Flynn S, Bloor CM (1989) Myocardial characteristics of thyroxine stimulated hypertrophy. A structural and functional study. Basic Res Cardiol 84: 345–358. 2530972 10.1007/BF02650869

[10] Adamson C, Maitra N, Bahl J, Greer K, Klewer S, et al (2004) Regulation of gene expression in cardiomyocytes by thyroid hormone and thyroid hormone analogs 3,5-diiodothyropropionic acid and CGS 23425 [N-[3,5-dimethyl-4-(4′-hydroxy-3′-isopropylphenoxy)-phenyl]-oxamic acid]. J Pharmacol Exp Ther 311: 164–171. 15148346 10.1124/jpet.104.069153

[11] De K, Ghosh G, Datta M, Konar A, Bandyopadhyay J, et al (2004) Analysis of differentially expressed genes in hyperthyroid-induced hypertrophied heart by cDNA microarray. J Endocrinol 182: 303–314. 15283691 10.1677/joe.0.1820303

[12] Bertin J, Guo Y, Wang L, Srinivasula SM, Jacobson MD, et al (2000) CARD9 is a novel caspase recruitment domain-containing protein that interacts with BCL10/CLAP and activates NF-kappa B. J Biol Chem. 275: 41082–41086. 10.1074/jbc.C000726200 11053425

[13] Perkins ND (1997) Achieving transcriptional specificity with NF-kappa B. Int J Biochem Cell Biol. 29: 1433–1448. 10.1016/s1357-2725(97)00088-5 9570137

[14] Misra A, Haudek SB, Knuefermann P, Vallejo JG, Chen ZJ, et al (2003) Nuclear factor-kappaB protects the adult cardiac myocyte against ischemia-induced apoptosis in a murine model of acute myocardial infarction. Circulation 108: 3075–3078. 14676146 10.1161/01.CIR.0000108929.93074.0B

[15] Sasaki H, Ray PS, Zhu L, Otani H, Asahara T, et al (2001) Hypoxia/reoxygenation promotes myocardial angiogenesis via an NF kappa B-dependent mechanism in a rat model of chronic myocardial infarction. J Mol Cell Cardiol 33: 283–294. 11162133 10.1006/jmcc.2000.1299

[16] Bell SE, Sanchez MJ, Spasic-Boskovic O, Santalucia T, Gambardella L, et al (2006) The RNA binding protein Zfp36l1 is required for normal vascularisation and post-transcriptionally regulates VEGF expression. Dev Dyn 235: 3144–3155. 17013884 10.1002/dvdy.20949

[17] Suri C, Jones PF, Patan S, Bartunkova S, Maisonpierre PC, et al (1996) Requisite role of angiopoietin-1, a ligand for the TIE2 receptor, during embryonic angiogenesis. Cell 87: 1171–1180. 8980224 10.1016/s0092-8674(00)81813-9

[18] Maisonpierre PC, Suri C, Jones PF, Bartunkova S, Wiegand SJ, et al (1997) Angiopoietin-2, a natural antagonist for Tie2 that disrupts in vivo angiogenesis. Science 277: 55–60. 9204896 10.1126/science.277.5322.55

[19] Sandhu R, Teichert-Kuliszewska K, Nag S, Proteau G, Robb MJ, et al (2004) Reciprocal regulation of angiopoietin-1 and angiopoietin-2 following myocardial infarction in the rat. Cardiovasc Res 64: 115–124. 15364619 10.1016/j.cardiores.2004.05.013

[20] Tuo QH, Zeng H, Stinnett A, Yu H, Aschner JL, et al (2008) Critical role of angiopoietins/Tie-2 in hyperglycemic exacerbation of myocardial infarction and impaired angiogenesis. Am J Physiol Heart Circ Physiol 294: H2547–2557. 18408125 10.1152/ajpheart.01250.2007

[21] Liu GY (2009) Isolation, sequence identification, and tissue expression profile of 3 novel porcine genes: NCF2, BCKDHB and BCKDHA. J Appl Genet 50: 47–50. 19193982 10.1007/BF03195651

[22] Matsuoka Y, Li X, Bennett V (2000) Adducin: structure, function and regulation. Cell Mol Life Sci 57: 884–895. 10950304 10.1007/PL00000731 PMC11146971

[23] Deacon DC, Nevis KR, Cashman TJ, Zhou Y, Zhao L, et al (2010) The miR-143-adducin3 pathway is essential for cardiac chamber morphogenesis. Development 137: 1887–1896. 20460367 10.1242/dev.050526

[24] Kahaly GJ, Dillmann WH (2005) Thyroid hormone action in the heart. Endocr Rev 26: 704–728. 15632316 10.1210/er.2003-0033

[25] van Rooij E, Olson EN (2007) MicroRNAs: powerful new regulators of heart disease and provocative therapeutic targets. J Clin Invest 117: 2369–2376. 17786230 10.1172/JCI33099 PMC1952642

[26] Callis TE, Pandya K, Seok HY, Tang RH, Tatsuguchi M, et al (2009) MicroRNA-208a is a regulator of cardiac hypertrophy and conduction in mice. J Clin Invest 119: 2772–2786. 19726871 10.1172/JCI36154 PMC2735902

[27] Ojamaa K (2010) Signaling mechanisms in thyroid hormone-induced cardiac hypertrophy. Vascul Pharmacol 52: 113–119. 20005976 10.1016/j.vph.2009.11.008 PMC2830872

[28] Zimmer HG, Gerdes AM, Lortet S, Mall G (1990) Changes in heart function and cardiac cell size in rats with chronic myocardial infarction. J Mol Cell Cardiol 22: 1231–1243. 2149393 10.1016/0022-2828(90)90060-f

[29] Gerdes AM, Moore JA, Hines JM, Kirkland PA, Bishop SP (1986) Regional differences in myocyte size in normal rat heart. Anat Rec 215: 420–426. 3740478 10.1002/ar.1092150414

[30] Pfeffer JM, Pfeffer MA, Braunwald E (1985) Influence of chronic captopril therapy on the infarcted left ventricle of the rat. Circ Res 57: 84–95. 3891127 10.1161/01.res.57.1.84

[31] Gentleman RC, Carey VJ, Bates DM, Bolstad B, Dettling M, et al (2004) Bioconductor: open software development for computational biology and bioinformatics. Genome Biol 5: R80. 15461798 10.1186/gb-2004-5-10-r80 PMC545600

[32] Kuhn K, Baker SC, Chudin E, Lieu MH, Oeser S, et al (2004) A novel, high-performance random array platform for quantitative gene expression profiling. Genome Res 14: 2347–2356. 15520296 10.1101/gr.2739104 PMC525694

[33] Lin SM, Du P, Huber W, Kibbe WA (2008) Model-based variance-stabilizing transformation for Illumina microarray data. Nucleic Acids Res 36: e11. 18178591 10.1093/nar/gkm1075 PMC2241869

[34] Du P, Kibbe WA, Lin SM (2008) lumi: a pipeline for processing Illumina microarray. Bioinformatics 24: 1547–1548. 18467348 10.1093/bioinformatics/btn224

[35] Smyth GK, Michaud J, Scott HS (2005) Use of within-array replicate spots for assessing differential expression in microarray experiments. Bioinformatics 21: 2067–2075. 15657102 10.1093/bioinformatics/bti270

[36] Smyth GK (2004) Linear models and empirical bayes methods for assessing differential expression in microarray experiments. Stat Appl Genet Mol Biol 3: Article3. 16646809 10.2202/1544-6115.1027

[37] Benjamini Y, Hochberg Y (1995) Controlling the false discovery rate: A practical and powerful approach to multiple testing. Journal of the Royal Statistical Society Series B (Methodological) 57: 289–300.

[38] Brazma A, Hingamp P, Quackenbush J, Sherlock G, Spellman P, et al (2001) Minimum information about a microarray experiment (MIAME)-toward standards for microarray data. Nat Genet 29: 365–371. 11726920 10.1038/ng1201-365

[39] Eisen MB, Spellman PT, Brown PO, Botstein D (1998) Cluster analysis and display of genome-wide expression patterns. Proc Natl Acad Sci U S A 95: 14863–14868. 9843981 10.1073/pnas.95.25.14863 PMC24541

[40] de Hoon MJ, Imoto S, Nolan J, Miyano S (2004) Open source clustering software. Bioinformatics 20: 1453–1454. 14871861 10.1093/bioinformatics/bth078

[41] Huang da W, Sherman BT, Lempicki RA (2009) Systematic and integrative analysis of large gene lists using DAVID bioinformatics resources. Nat Protoc 4: 44–57. 19131956 10.1038/nprot.2008.211

[42] Huang da W, Sherman BT, Lempicki RA (2009) Bioinformatics enrichment tools: paths toward the comprehensive functional analysis of large gene lists. Nucleic Acids Res 37: 1–13. 19033363 10.1093/nar/gkn923 PMC2615629

